# Data sets of measured pantograph voltage and current of European AC railways

**DOI:** 10.1016/j.dib.2020.105477

**Published:** 2020-04-20

**Authors:** Andrea Mariscotti

**Affiliations:** DITEN, University of Genova, Via Opera Pia 11A, I-16145 Genova, Italy

**Keywords:** Harmonics, Measurement of electrical quantities, Power quality, Railways, Rolling stock

## Abstract

AC railways are characterized by peculiar Power Quality phenomena, where moving loads (trains, locomotives, etc.) interact with the supply network that provides electrical energy through the overhead contact line. Distortion, resonances, transients overlap in a complex dynamic scenario, that sees several and various problems of Power Quality, network stability, power and energy metering and disturbance to systems and equipment. For all related studies and analysis raw experimental data are extremely important. The provided data consists of time-domain waveforms of sampled pantograph voltage and current: each recording is tagged with the specific train operating condition (traction, cruising/coasting, braking, standstill), the active power and the speed, to support correlation and clustering of data.

Specifications table

**Table:** 

Subject	Electrical and Electronic Engineering
Specific subject area	Measurement of electrical quantities, Power Quality
Type of data	Table Graph Figure Raw data provided
How data were acquired	Digitized sampled data using various data recorders located onboard and connected to voltage and current sensors (voltage dividers and Rogowski coils). Data processing for figures and tables is by means of own Matlab scripts, validated through many years of comparison with other sources.
Data format	Raw (sampled waveforms in Matlab .mat binary file, double precision) analysed (post-processed data in figures and tables)
Parameters for data collection	Data acquisition and digitization with 50 kSa/s sample rate, 16 bit resolution, streaming to disk. Pantograph quantities sensed with transducers whose uncertainty is included. Trains either in commercial service or dedicated for tests, in normal operating conditions.
Description of data collection	A train in normal commercial service was used in each respective country with the acquisition system and sensors installed onboard. The acquisition was run for some days and then data downloaded when the data recorder was uninstalled and returned. Additional train variables, such as speed and location, were also acquired. Speed is also reported for better characterization of the data.
Data source location	1) Zurich-Brig railway line (Switzerland), 15 kV 16.7 Hz railway system 2) Rome-Naples railway line (Italy), 2 × 25 kV 50 Hz railway system 3) Hamburg – Dortmund – Frankfurt railway line (Germany), 15 kV 16.7 Hz railway system 4) Paris – Lyon railway line (France), 2 × 25 kV 50 Hz railway system
Data accessibility	- With the article (for processed data appearing in figures and tables) - In public repository (for raw data): Repository name: Mendeley Data Data identification number: 10.17632/yscdzrn45y.1 Direct URL to data: http://data.mendeley.com/datasets/yscdzrn45y/draft?a=60e77129-6a90-4ae9-a1f2-4b8bfe27aac3

## Value of the data

• Recordings of pantograph electrical quantities are difficult to obtain from railway operators for safety and organizational issues, as well as a general data protection concern.
• The dataset may be used for Power Quality studies, to analyse the electrical behaviour of the traction supply system – rolling stock interaction
• The dataset may be used as well to synthesize a source of emissions for simulation studies and to assess distortion against limit masks for interference to railway signalling.
• The size of the dataset (21,033 short recordings overall) ensures statistically significant sets of data, spread over various rolling stock operating conditions and traction line impedance values.
• Data can be used by researchers and students, as a comprehensive set representative of the electrical behaviour of four major ac railways in Europe.

## 1 Data description

The raw data consist of pantograph voltage (*v_p_*) and current (*i_p_*) recordings, organized in short records (we name “snippets”) of 5 fundamental cycles. Each snippet is tagged with information regarding: the rolling stock operating condition (ACCeleration, BRaKing and STandStill, where in capital letters we have indicated convenient three-letter tags, coded then numerically as +1, −1 and 0 for an exigency of compactness), the rms intensity (indicating the overall amount of power absorbed or regenerated), and the speed. Another operating condition related to coasting can be recovered by the user, simply checking low current absorption and non-null speed (possibly constant or slightly decaying across adjacent snippets)

So, the elementary data structure consists of a pair of vectors (the two snippets *v_p_*(*n*) and *i_p_*(*n*)) and three numeric fields (the “tag” from −1 to +1, the rms current intensity *Irms*(*n*) and the speed *s*(*n*)).

The snippets are arranged and stored using Matlab data structures: “vp” and “ip” are matrices of size RxN, where R is the number of collected snippets and N is the number of samples in a snippet; a Rx3 vector side the raw data with the three components made of “tag”, “Irms” and “s”. Then, support quantities are added: “fs” is the sampling frequency of 50 kHz and “fund” is the nominal fundamental frequency of the system.

This structure is created for each of the four railway systems and stored in four separate files: France.mat, Germany.mat, Italy.mat and Switzerland.mat. The size of the four datasets consists of 5337 (Switzerland), 3554 (Germany), 3809 (Italy) and 4258 (France) snippets (5-cycle records), together with their tagging information.

**Fig. 1:**
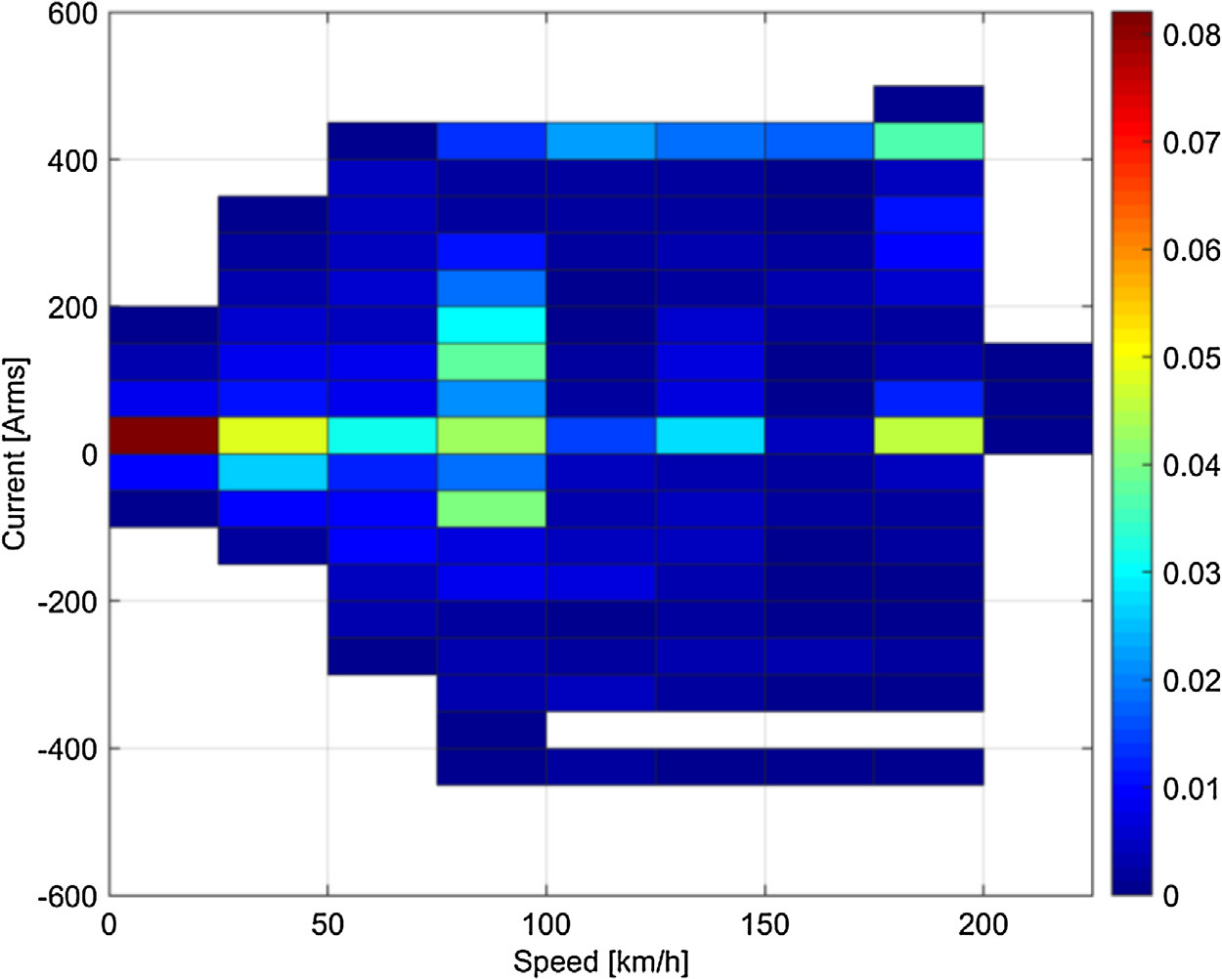
Statistical distribution of speed and rms current for the runs on the 16.7 Hz system (Switzerland).

**Fig. 2:**
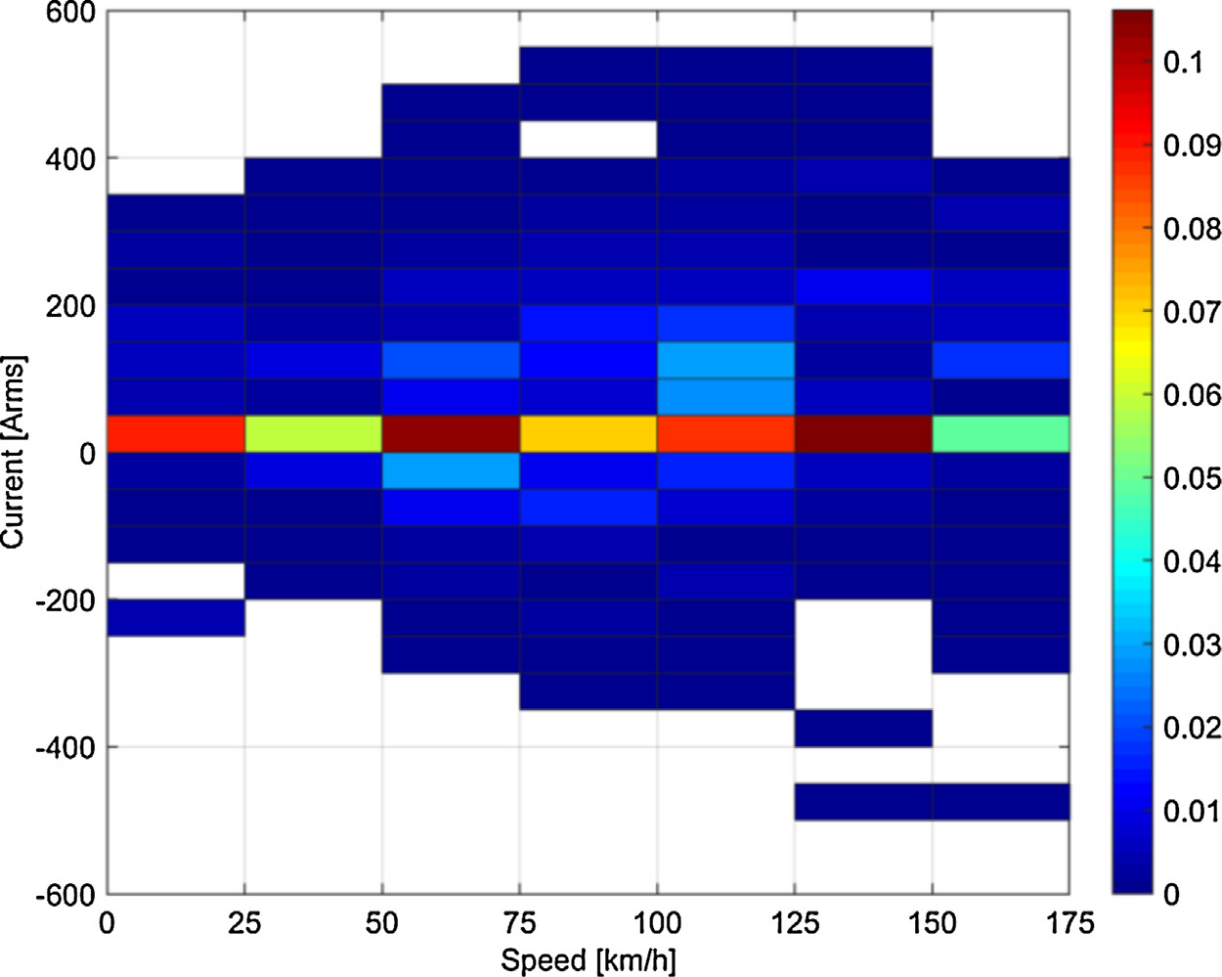
Statistical distribution of speed and rms current for the runs on the 16.7 Hz system (Germany).

**Fig. 3:**
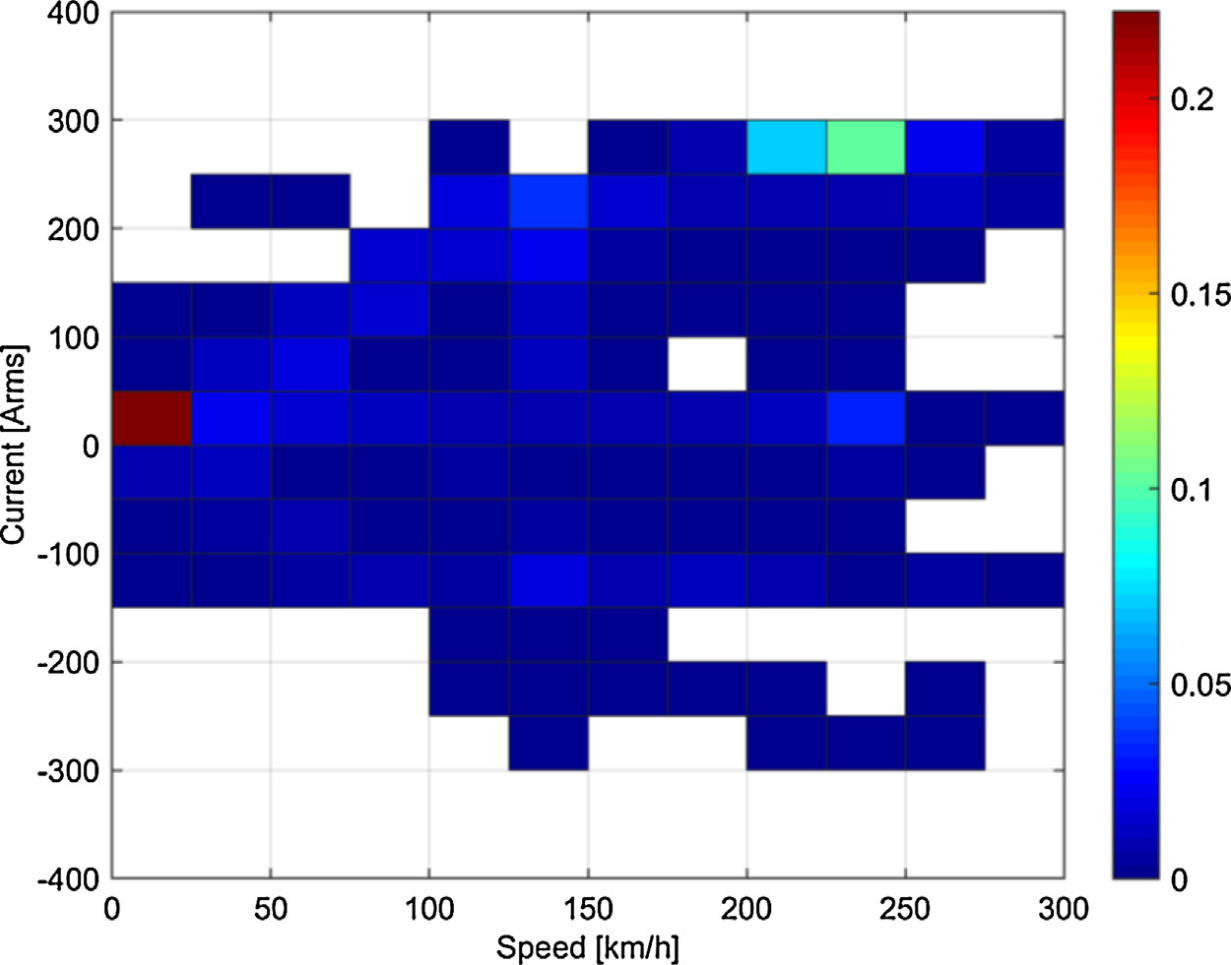
Statistical distribution of speed and rms current for the runs on the 50 Hz system (Italy).

**Fig. 4:**
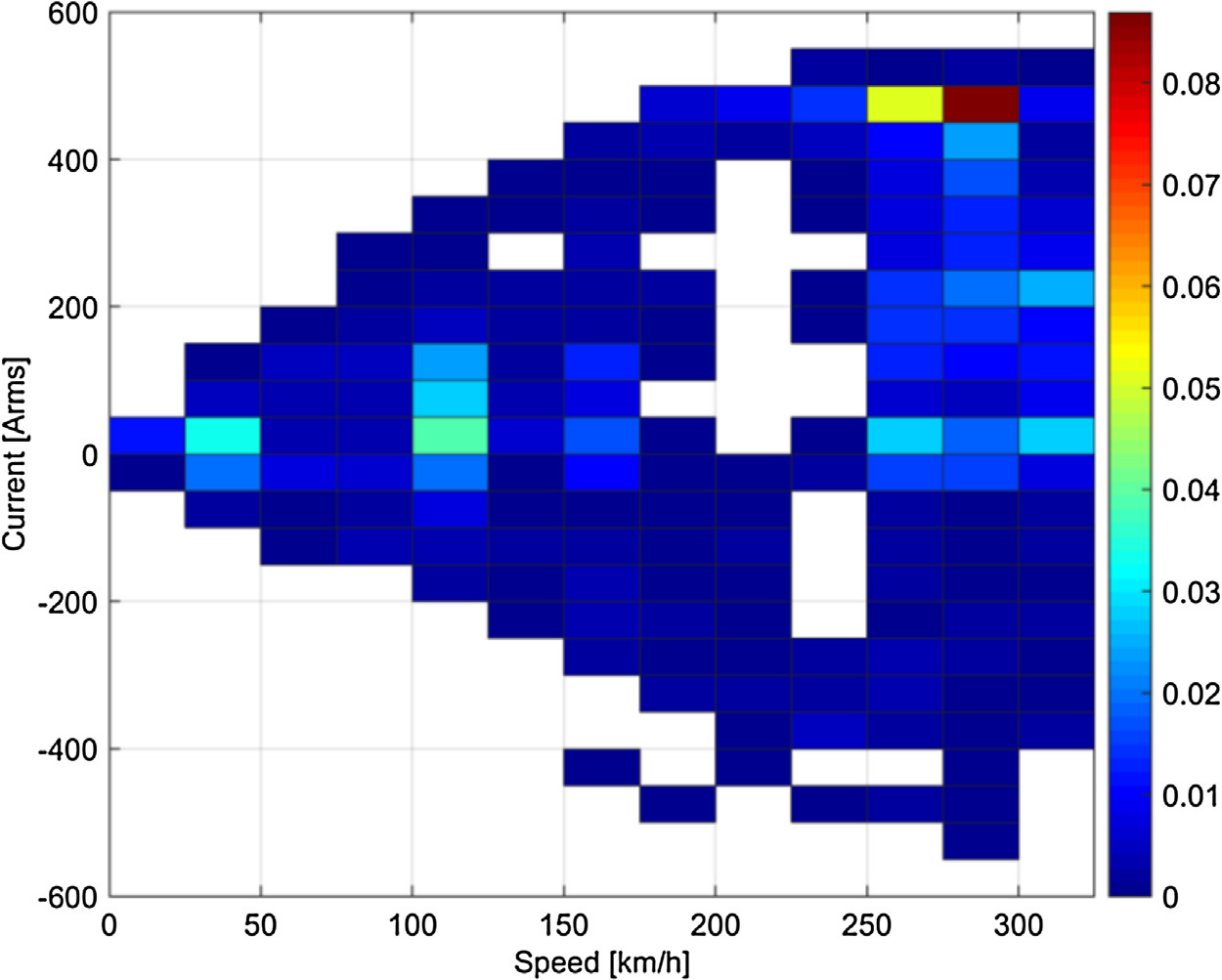
Statistical distribution of speed and rms current for the runs on the 50 Hz system (France).

The statistical distributions of speed and rms current tag values (including the sign of the current to distinguish between traction and braking phases) are shown in Fig. 1, Fig. 2, Fig. 3, Fig. 4.

**Fig. 5:**
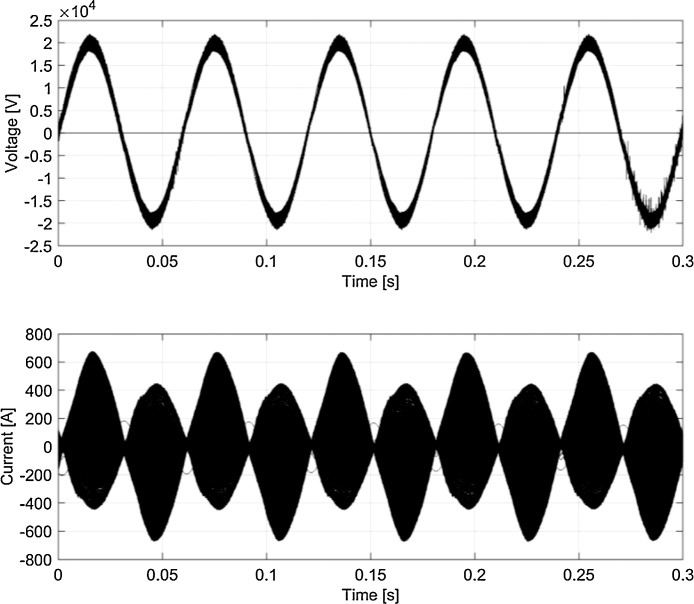
Typical waveforms (persistency plot) for *v_p_* and *i_p_* (Switzerland, 16.7 Hz system).

**Fig. 6:**
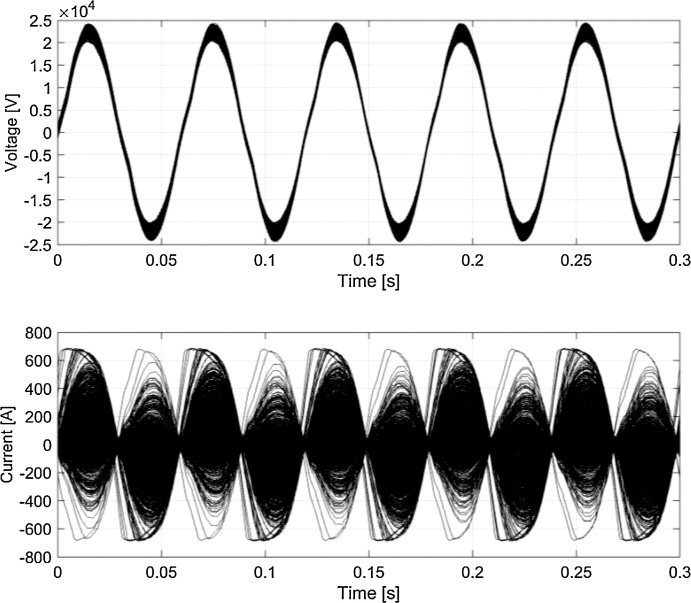
Typical waveforms (persistency plot) for *v_p_* and *i_p_* (Germany, 16.7 Hz system).

**Fig. 7:**
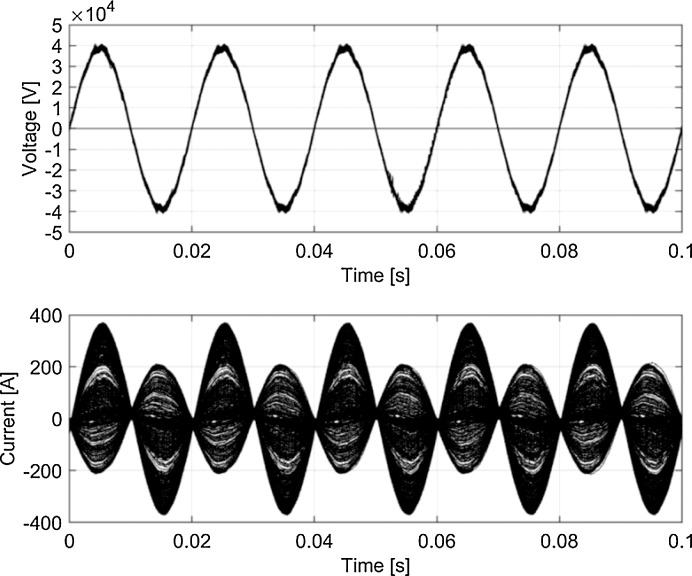
Typical waveforms (persistency plot) for *v_p_* and *i_p_* (Italy, 50 Hz system).

**Fig. 8:**
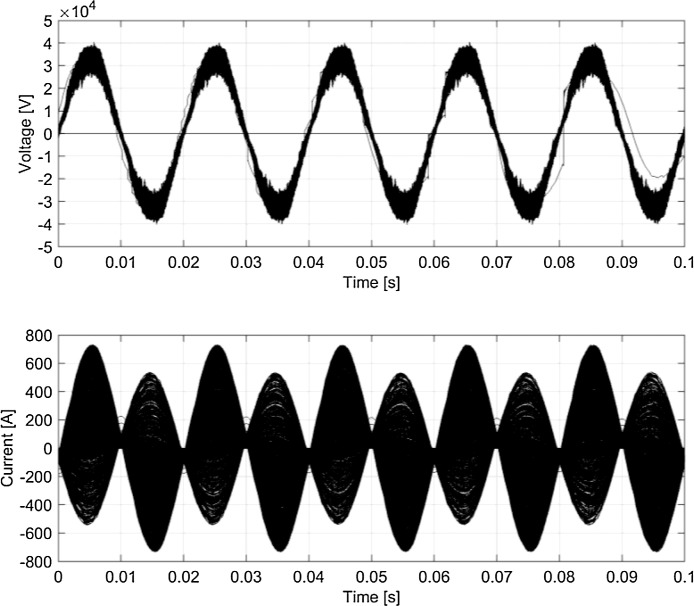
Typical waveforms (persistency plot) for *v_p_* and *i_p_* (France, 50 Hz system).

The typical waveforms are shown in Fig. 5, Fig. 6, Fig. 7, Fig. 8 using persistency plots.

**Fig. 9:**
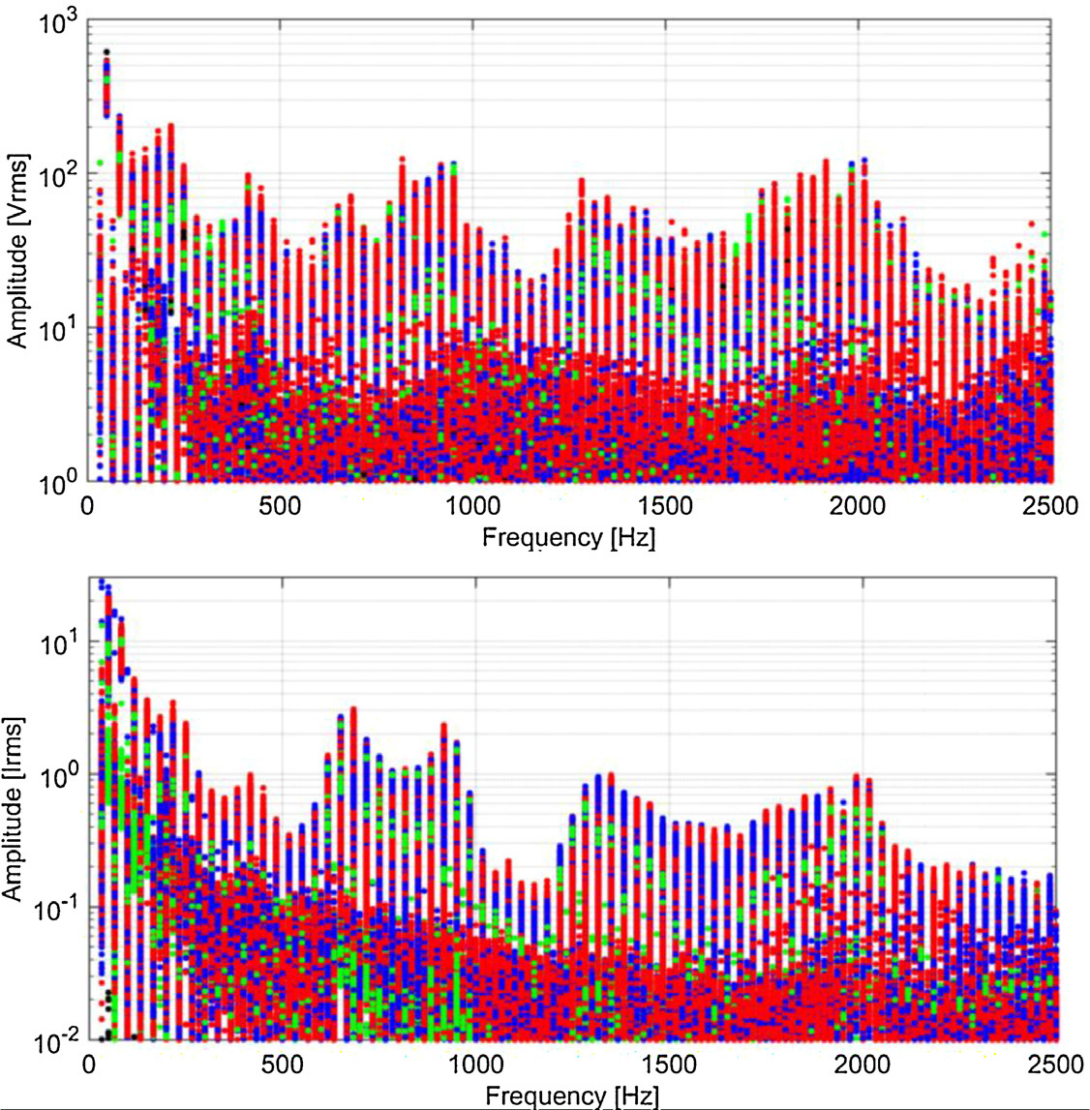
Typical spectra of *V_p_* and *I_p_*, colour coded for traction (red), braking (blue), coasting (black) and standstill (green) (Switzerland, 16.7 Hz system). (For interpretation of the references to colour in this figure legend, the reader is referred to the web version of this article.)

**Fig. 10:**
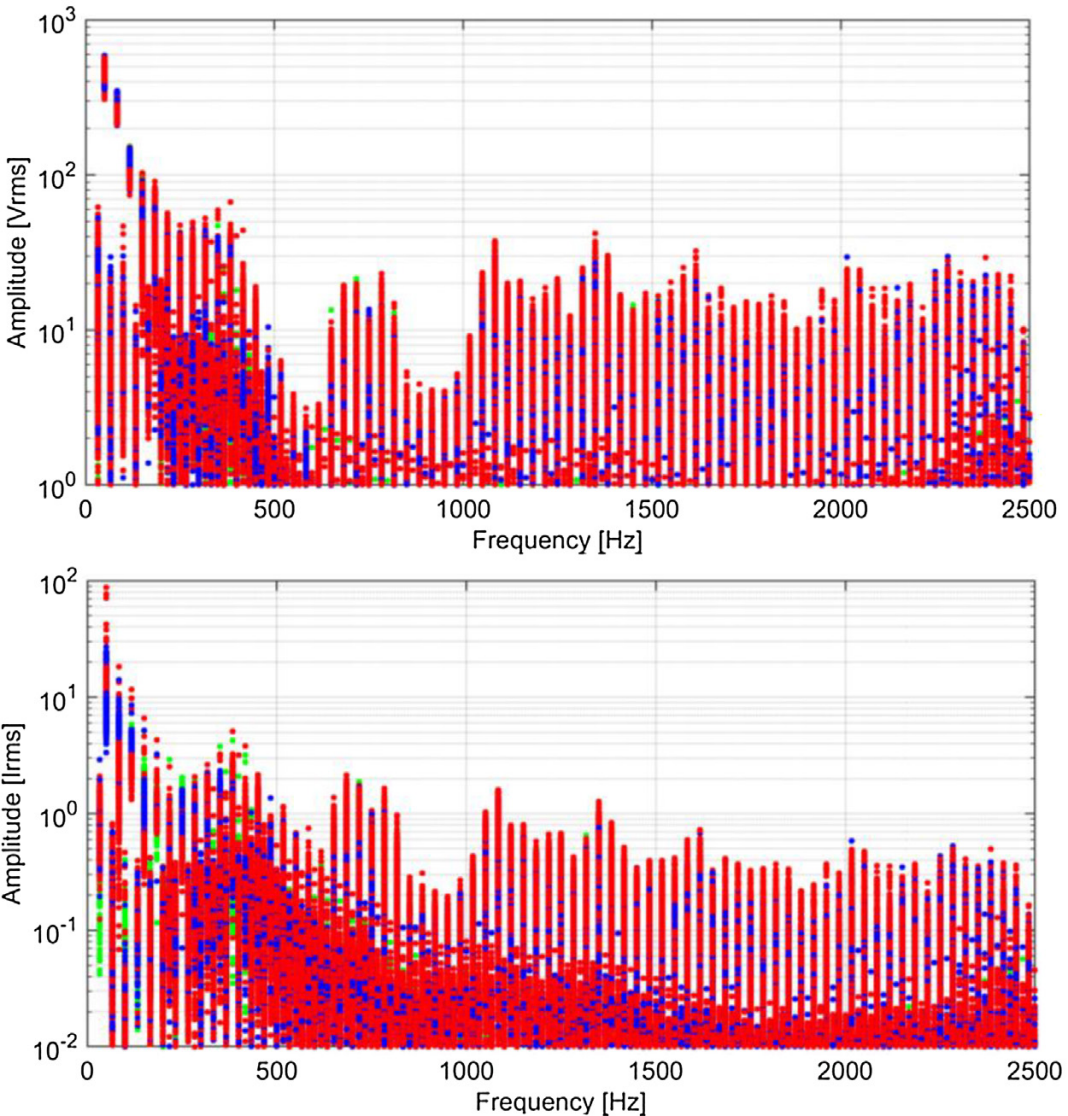
Typical spectra of *V_p_* and *I_p_*, colour coded for traction (red), braking (blue), coasting (black) and standstill (green) (Germany, 16.7 Hz system). (For interpretation of the references to colour in this figure legend, the reader is referred to the web version of this article.)

**Fig. 11:**
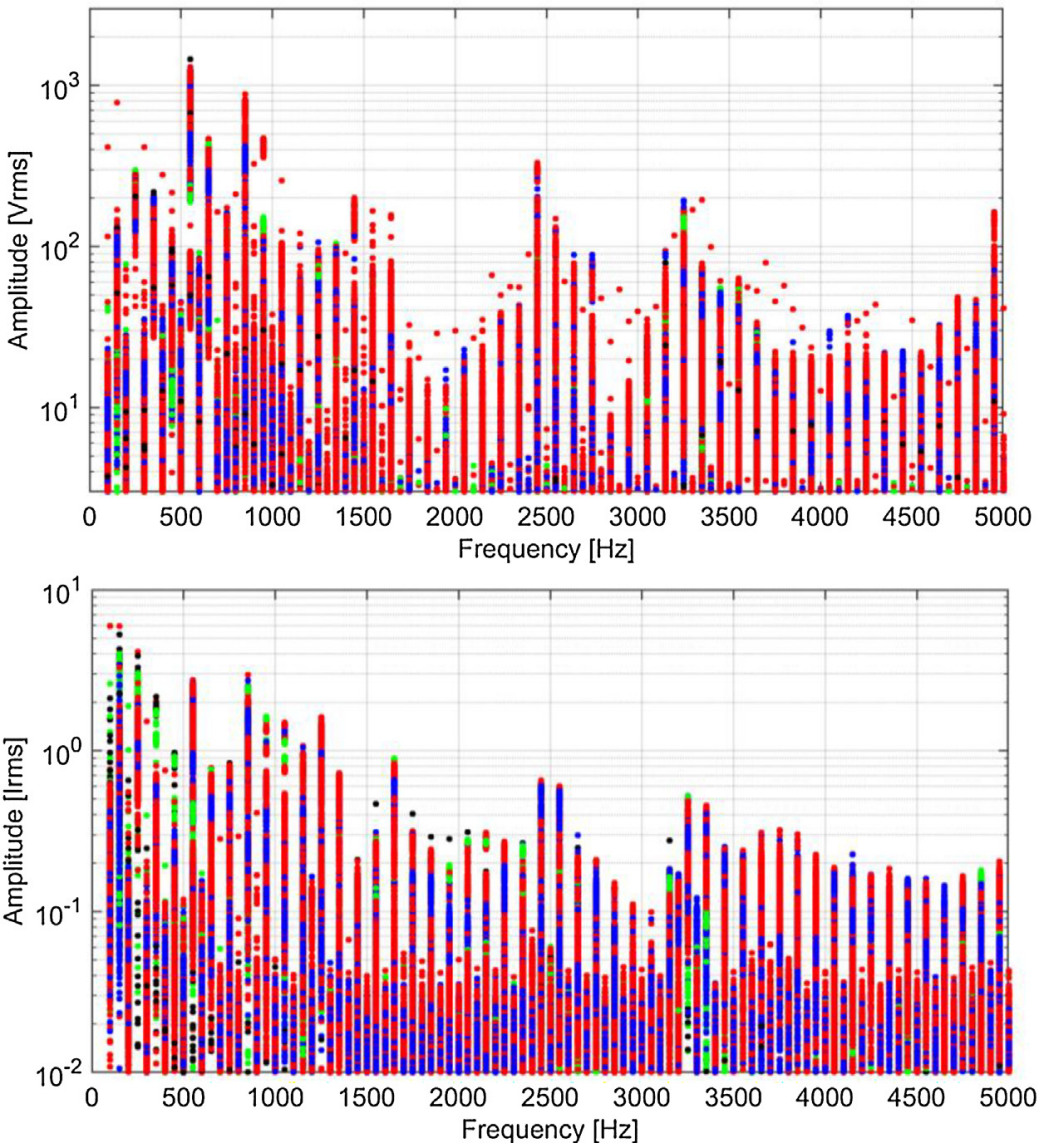
Typical spectra of *V_p_* and *I_p_*, colour coded for traction (red), braking (blue), coasting (black) and standstill (green) (Italy, 50 Hz system). (For interpretation of the references to colour in this figure legend, the reader is referred to the web version of this article.)

**Fig. 12:**
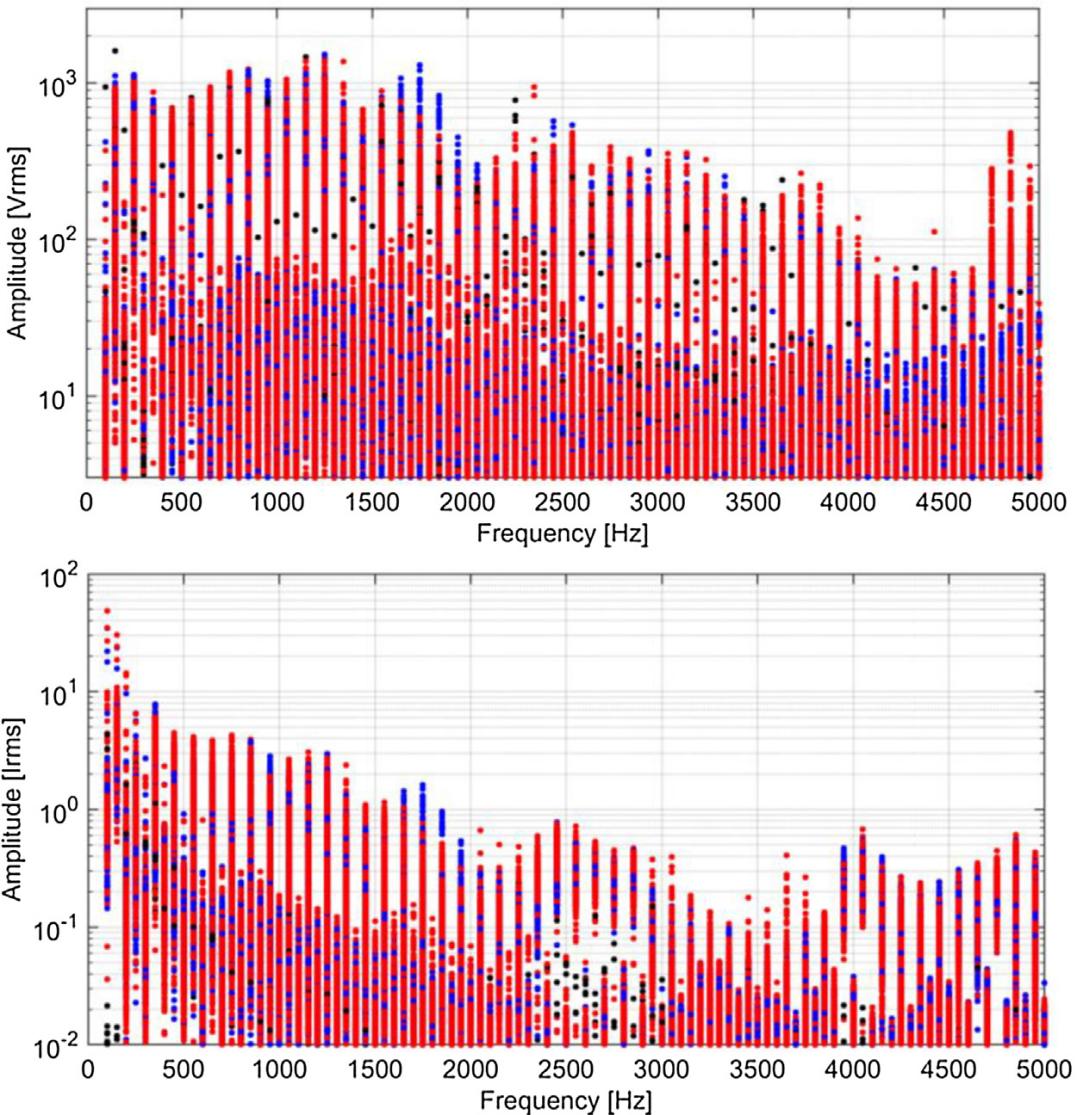
Typical spectra of *V_p_* and *I_p_*, colour coded for traction (red), braking (blue), coasting (black) and standstill (green) (France, 50 Hz system). (For interpretation of the references to colour in this figure legend, the reader is referred to the web version of this article.)

Spectra are shown in Fig. 9, Fig. 10, Fig. 11, Fig. 12 using a graphical representation that separates traction, braking, coasting and standstill conditions, and focuses on harmonics of the fundamental, discarding the other components that are spaced by one fifth of the fundamental, having used the entire 5 cycles for the calculation.

**Fig. 13:**
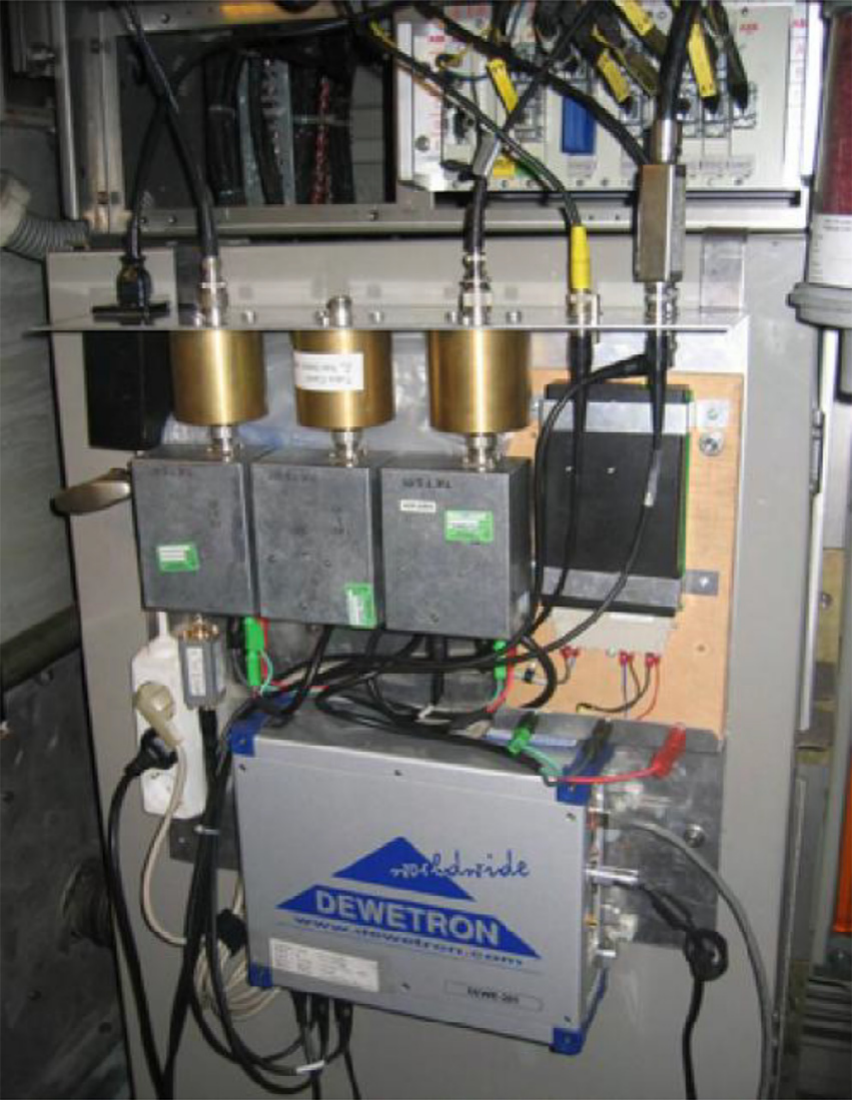
Example of arrangement of the data acquisition setup for the measurements in France, Germany and Switzerland.

The arrangement of the data acquisition system used in France, Germany and Switzerland is shown in Fig. 13.

Some clustering of speed-current data may be observed, depending on the type of tests or service: for example, looking at Fig. 3 braking in Italy was particularly intense in the initial phase at high speed, so that pairs of 200–250 km/h speed and 250–300 Arms current have higher probability; conversely, the Swiss loco in normal service has a more dispersed and “natural” distribution of speed and current values (see Fig. 1).

In general we took care to discard data with aberrations and significant transients by applying a set of verification rules based on the compliance to the EN 50163 voltage levels [1], on skipping neutral sections and consequential transients, on limiting data recorded during train stops (that for trains used only for tests might have been much longer than normal service stops, as it was indeed).

## 2 Experimental design, materials, and methods

The pantograph voltage (*v_p_*) and current (*i_p_*) recordings were taken during long test runs in 2008 on the railway lines of four different European traction supply systems using various types of rolling stock (locomotives and electrical multiple units, EMUs), one in commercial service (Switzerland) and three dedicated to the tests (France, Germany and Italy):

• the Zurich-Brig line in Switzerland, featuring a 15 kV 16.7 Hz supply and using a Re460 locomotive with 5.6 MW nominal power;
• the Hamburg – Dortmund – Frankfurt line in Germany, featuring a 15 kV 16.7 Hz supply and using a ICE EMU with 9.6 MW total nominal power;
• the Rome-Naples line in Italy, featuring a 2 × 25 kV 50 Hz supply and using an ETR500 EMU with 8.8 MW total nominal power;
• the Paris – Lyon line in France, featuring a 2 × 25 kV 50 Hz supply and using a TGV Dasye EMU with 9.28 MW total nominal power.

The main characteristics and the architectures of the 16.7 Hz and 50 Hz railway systems are described in [2,3].

The measurements were possible thanks to the participation to the Railcom project of several manufacturers, train operators and infrastructure owners. This kind of measurements necessitates a great deal of planning and organization, as well as a demonstration of the reliability of the measurement system and its installation, to clear doubts about impact on safety, traffic and timetable disruption.

The dataset covers two quite different ac railway system architectures (16.7 Hz and 50 Hz systems).

Data were sampled each at 50 kSa/s with 16 bit resolution data recorders in 2008 and stored in high-precision data format. The pantograph voltage and current were sensed with various transducers:

• for France, Germany and Switzerland [4]: a capacitive voltage divider calibrated each time is installed for the unavoidable geometry tolerances, and a commercial Rogowski coil (Fluke, mod. R3010);
• for Italy the Trenitalia measurement setup was used, adopting a current transformer instead of Rogowski coil [5].

Some factors with metrological relevance were taken into account when devising and installing the measurement system:

• electrical noise on the connection cables and equipment, attenuated by using equipotential metallic frame, low-impedance bonding and filters (shown in Fig. 9) and verified by initial manned acquisition for quantification of noise and offsets, at each new installation;
• location of sensors was optimized, as for Rogowski coils, centred on the high-voltage cables branching from the pantograph to minimize uncertainty;
• a check was done at the beginning of each test campaign, including the calibration of the voltage divider for Switzerland, Germany and France.

The attached dataset (link reported in Appendix A) consists of 5-cycle snippets: the time duration is 5*T*, where *T* is the fundamental period of 60 and 20 ms, respectively for the 16.7 Hz and 50 Hz systems. These data were extracted from continuous recordings taken when running on the said lines.

The data lend themselves to be processed with Fourier analysis using a Discrete Fourier Transform (DFT) approach. To this aim attention must be paid to possible discontinuity at the two extremes of each snippet if implicitly periodized as for DFT, because of, first, some underlying slow fluctuations of the quantities (e.g. caused by the variable loading of the traction line), and, second, the variability of the instantaneous frequency (important for high-order harmonics, but quite limited anyway over a 10-cycle snippet; it is documented in [6,7] for the 16.7 Hz and 50 Hz systems). The use of tapering windows is strongly advised.

Fig. 5–8 report the overall set of 5-cycle snippets for each railway system plotted using persistency in order to show the typical dispersion of data in time domain.

These data have been used in the past to extract the following type of information and subject to the following type of analysis:

• calculation of the harmonic distortion of the system, identifying characteristic harmonics and their variability and statistics [8];
• evaluation of energy efficiency in railways, by accounting for harmonic active and non-active power [9,10];
• assessment of harmonic power indicators with a single-point metering approach [11].

Please, see cited references for methods, results and additional bibliography.

## Appendix Supplementary material

Image, application 1
